# Discovering Che-1/AATF: a new attractive target for cancer therapy

**DOI:** 10.3389/fgene.2015.00141

**Published:** 2015-04-10

**Authors:** Simona Iezzi, Maurizio Fanciulli

**Affiliations:** Laboratory of Epigenetics, Molecular Medicine Area, Regina Elena National Cancer Institute, RomeItaly

**Keywords:** Che-1/AATF, DNA damage response, transcription, apoptosis, cell cycle regulation, cellular stress, survival

## Abstract

The transcriptional cofactor Che-1/AATF is currently emerging as an important component of the DNA damage response (DDR) machinery, the complex signaling network that maintains genome integrity and prevents tumorigenesis. Moreover this protein is involved in a wide range of cellular pathways, regulating proliferation and survival in both physiological and pathological conditions. Notably, some evidence indicates that dysregulation of Che-1/AATF levels are associated with the transformation process and elevated levels of Che-1/AATF are required for tumor cell survival. It is for these reasons that Che-1/AATF has been regarded as an attractive, still theoretical, therapeutic target for cancer treatments. In this review, we will provide an updated overview of Che-1/AATF activities, from transcriptional regulation to DDR.

## Che-1/AATF at a Glance

More than 10 years ago human Che-1/AATF was identified by two different groups as both an RNA polymerase II binding protein and a gene downregulated upon TGF β induced differentiation (Fanciulli et al., 2000; Lindfors et al., 2000).

The human *Che-1/AATF* gene is located on chromosome 17, a region of the genome that is very rich in protein-coding genes, segmental duplications, and home to genes implicated in a wide range of human genetic diseases, such as *BRCA1* and *TP53* (Zody et al., 2006). It is highly conserved among eucaryotes and encodes for a protein of 558 aminoacids, whose expression is regulated by a negative feedback mechanism in which Che-1/AATF is present on its own promoter exerting an inhibitory effect (Monaco et al., 2003). At the structural level, the protein is characterized by the presence of an N-terminal acidic domain, a canonical leucine zipper, and three LXXLL motifs for nuclear receptor binding. It also contains two nuclear and two putative nucleolar localization signals (Fanciulli et al., 2000; Lindfors et al., 2000; Scott et al., 2011) and, at the cellular level Che-1/AATF mostly shows a nuclear and nucleolar localization. However, a cytoplasmic localization has also been reported in primary cerebellar granule neurons (Barbato et al., 2003; Di Certo et al., 2007), hippocampal neurons (Guo and Xie, 2004), and mouse embryonic fibroblasts (Höpker et al., 2012).

## Che-1/AATF is a Transcriptional Cofactor

Che-1/AATF’s ability to bind the RNA polymerase II in addition to the observation that its rat ortholog exhibits transactivation activity (Page et al., 1999), suggested from the beginning that Che-1/AATF could have been a transcriptional cofactor involved in the regulation of gene expression by connecting specific transcription factors to the general transcriptional machinery. In fact, Che-1/AATF has been shown to interact with nuclear hormone receptors *in vitro* and to enhance transactivation of several steroid hormone receptors, alone or in cooperation with histone acetyltransferase p300 (Leister et al., 2003). In addition, it has also been reported that Che-1/AATF activity on androgen receptor mediated transcription is enhanced by its interaction with the tumor suppressor protein TSG101 (Burgdorf et al., 2004). Up until now, in addition to nuclear hormone receptors, several transcription factors, including the retinoblastoma protein (pRb), p65, and STAT3 (Bruno et al., 2002, 2006; Ishigaki et al., 2010), have been proven to interact with Che-1/AATF, thereby involving it in multiple cellular processes. These interactions are mostly regulated by post-translational modifications, which provide a rapid and reversible manner to modulate Che-1/AATF co-transcriptional activity in response to different stimuli (**Table 1**). In this regard, it is interesting to note that this protein interacts with RNA polymerase II through the C-terminal region of the subunit 11 (hRPB11; Fanciulli et al., 2000). This subunit is encoded by a multigene family which produces, along with the main form hRPB11a, proteins differing in their C-terminal domain, with different binding abilities and differently expressed in several tissues (Grandemange et al., 2001; Benga et al., 2005). Thus, Che-1/AATF action on transcription may depend on its binding of both transcription factors or different forms of hRPB11.

**Table 1:** Che-1/AATF post-translational modifications.

Modification	Residue	Enzyme	Function	Reference
Phosphorylation	S181	ATM	Stabilization upon DNA damage; modulation of protein–protein interactions	Bruno et al. (2006)
	S141 S474 S508	Chk2	Stabilization upon DNA damage; modulation of protein–protein interactions	Bruno et al. (2006)
	T144	HIPK2	Degradation following apoptotic DNA damage	De Nicola et al. (2014)
	S316* S320* S321*		Modulated upon autophagy inhibition	Alayev et al. (2014)
	T366*	MK2	Nuclear translocation	Höpker et al. (2012)
		Cdk5		Buontempo et al. (2008)
Poly(ADP ribosyl)ation		PARP-1	Stabilization upon DNA damage	Bacalini et al. (2011)
Ubiquitination		HDM2	Degradation following apoptotic DNA damage	De Nicola et al. (2007)
Isomerization	P145	Pin1	Prerequisite for ubiquitination and degradation	De Nicola et al. (2007)

Che-1 and AATF genes were cloned independently by two different groups. They encode for the same protein but in GenBank their sequences differ in the total number of aminoacids. AATF is reported as a protein composed of 560 aa while Che-1 of 558 aa. In order to avoid confusion we reported the position of the modified residue as indicated in the literature and the sequence which is referred to. *indicates AATF sequence.

## Che-1/AATF in Proliferation and Cell Cycle Control

Che-1/AATF protein is ubiquitously expressed (Fanciulli et al., 2000; Lindfors et al., 2000) and its expression is essential for proliferation and survival since Traube (Che-1/AATF mouse ortholog) knock out mice are embrionically lethal at the preimplantation state (Thomas et al., 2000). Moreover, mutant embryos exhibit a significant reduction in the total number of cells, indicating Che-1/AATF’s involvement in cell cycle regulation. Consistent with these data, Bruno et al. (2002) demonstrated that Che-1/AATF promotes cell cycle progression by inhibiting the growth suppression functions of the pRb protein. pRb exerts its anti-proliferative functions by interacting with transcription factors E2F and promoting the assembly of an inhibitory complex containing histone deacetylases (i.e., HDAC1) on the promoters of E2F-responsive genes, whose expression is essential for the transition G1/S (Dick and Rubin, 2013). Che-1/AATF directly binds pRB and removes HDAC1 from the Rb/E2F complex, allowing transcription and progression to the S phase (Bruno et al., 2002). This activity may be modulated by its interaction with IFT88/polaris, a centrosomal protein that negatively regulates G1–S transition and inhibits Che-1/AATF binding to pRb (Robert et al., 2007). Remarkably, Che-1/AATF is hyperphosphorylated and accumulated during the G1/S transition (Bruno et al., 2002), suggesting that post-translational modifications may also regulate Che-1/AATF pro-proliferative functions. In addition, it has been recently shown that Che-1/AATF also participates in the control of mitotic entry by localizing at interphase centrosomes and regulating centrosome duplication and spindle formation (Sorino et al., 2013).

## Che-1/AATF is an Anti-Apoptotic Factor

Along with its pro-proliferative role, Che-1/AATF also exhibits strong anti-apoptotic activity. Indeed, the rat AATF protein was originally identified for its ability to interact with and antagonize the activity of Dlk/ZIP (ZIPK), a serine/threonine kinase involved in the induction of apoptosis (Page et al., 1999).

Up until now much of the information regarding the anti-apoptotic function of Che-1/AATF derives from studies performed in the neural tissue, where this protein seems to be involved in the regulation of the apoptotic signaling in both physiological and pathological conditions. Di Certo et al. (2007) showed a direct interaction between Che-1/AATF and neurotrophilin receptor interacting MAGE homolog “NRAGE,” an inducer of cell-death during neuronal development. In particular, they demonstrated that Che-1/AATF counteracts NRAGE-induced apoptosis, while NRAGE overexpression induces Che-1/AATF degradation by targeting it to the ubiquitin–proteasome pathways (Di Certo et al., 2007). Similarly, some evidence suggests that Che-1/AATF anti-apoptotic activity is involved in the neurodegeneration process associated with neurodegenerative diseases, such as Alzheimer’s. This pathology is associated with extracellular aggregates of the β-amyloid peptide (Aβ) and intraneuronal fibrillar tangles of the microtubule binding protein Tau (Crews and Masliah, 2010). It has been demonstrated that Che-1/AATF can counteract neuronal degeneration induced by Aβ by interacting with prostate apoptosis response-4 (par-4) and blocking the par-4 mediated aberrant production and secretion of the neurotoxic peptide (Xie and Guo, 2004). Moreover, Che-1/AATF interacts with Tau in rat cerebellar granule neurons where this interaction is modulated during neuronal apoptosis (Barbato et al., 2003). A further indication of Che-1/AATF involvement in neurodegeneration is the demontration that it interacts with and is a substrate of cyclin-dependent kinase 5 (Cdk5), a serine/threonine protein kinase, whose activity is deregulated in neurodegenerative diseases (Buontempo et al., 2008).

A protective role of Che-1/AATF has also been reported in human kidney proximal tubule cells, where this protein has been observed to counteract apoptotic cell death following induced-renal injury by preserving mitochondrial function and reducing oxidative damage (Xie and Guo, 2006).

However, a pro-apoptotic role of Che-1/AATF has been recently reported. Ferraris et al. (2012) demonstrated that Che-1/AATF overexpression enhances UV induced apoptosis by promoting phosphorylation and transactivational activity of the pro-apoptotic factor cJun, in a p53 independent manner. Moreover, UV damage induces Che-1/AATF redistribution from nucleolus to nucleoplasm, thus allowing a direct Che-1/AATF-cJun interaction (Ferraris et al., 2012).

## Che-1/AATF is Involved in the Cellular Response to Different Kind of Stress

### DNA Damage

An increasing number of studies indicate Che-1/AATF as an important component of the DNA damage response (DDR), a complex network of pathways that eucaryotic cells have evolved to maintain genome integrity and prevent tumorigenesis (Jackson and Bartek, 2009; Lord and Ashworth, 2012). DDR coordinates multiple factors that cooperate together to detect genomic lesions, arrest cell cycle in order to allow repair, and promote apoptosis or senescence if damage is too severe (Ciccia and Elledge, 2010).

Upon DNA damage Che-1/AATF is extensively modified by post-translational modifications affecting its localization, half-life and interacting partners. It has been demonstrated that checkpoint kinases MK2, ATM, Chk2 can phosphorylate and activate this protein (Höpker et al., 2012; Bruno et al., 2006). Höpker et al. (2012) have shown that DNA damage promotes Che-1/AATF phosphorylation by checkpoint kinase MK2 at residue T366. This modification induces translocation of Che-1/AATF from the cytoplasm to the nucleus where it inhibits transcription of p53 dependent proapoptotic genes, such as *Puma*, *Bax*, and *Bak* (Höpker et al., 2012). On the other hand, phosphorylation through ATM and Chk2 leads to Che-1/AATF stabilization and accumulation by increasing its resistance to proteasome degradation (Bruno et al., 2006). Moreover, these latter modifications greatly affect Che-1/AATF functions, acting as a molecular switch that moves this protein from the pathways regulating cell cycle progression to the ones involved in cell cycle arrest and survival. In particular, (ATM–Chk2) phosphorylated-Che-1/AATF relocates from E2F1-dependent promoters to the promoters of genes involved in checkpoint activation such as *TP53* and *p21*, thus allowing their transcription and cell cycle arrest at the G2/M checkpoint (Bruno et al., 2006). Interestingly, these modifications also promote a specific interaction between Che-1/AATF and tumor suppressor p53. This binding occurs at the early stage of the DDR and specifically directs p53 toward the transcription of genes involved in cell cycle arrest. Notably, the two proteins detach when DNA damage is not repairable and cells undergo apoptosis (Desantis et al., 2015a). Evidence also shows that upon DNA damage phosphorylated-Che-1/AATF, by ATM and Chk2, promotes the transcription of the anti-apoptotic factor XIAP, an inhibitor of caspase activity. Consistent with this observation, Che-1/AATF overexpression protects cells from apoptosis induced by DNA damaging agents (Bruno et al., 2008).

It is worth remembering that, other than phosphorylation, poly(ADP-ribosyl)ation also participates in regulating Che-1/AATF activities upon genotoxic stress. In fact, it has been demonstrated that poly (ADP-ribose) polymerase 1 (PARP-1) interacts with this protein and promotes its modification, which in turn contributes to Che-1/AATF stabilization upon DNA damage (Bacalini et al., 2011).

A recent study revealed that Che-1/AATF is also part of the spindle assembly checkpoint (SAC), a ubiquitous safety mechanism that ensures the fidelity of chromosome segregation during mitosis and cooperates with the proteins of the DDR network in restricting mitotic progression in response to DNA damage (Zhang et al., 2007; Lara-Gonzalez et al., 2012). In particular, it was shown that DNA damage induces centrosomal accumulation of Che-1/AATF and depletion of this protein is associated with an increase in the number of centrosomes, multipolar spindles, failure to arrest mitosis, and apoptosis in response to genotoxic treatments (Sorino et al., 2013).

In agreement with its anti-apoptotic and prosurvival roles, Che-1/AATF degradation is required to execute the apoptotic program when DNA damage is too severe and cannot be repaired. The complex signaling cascade that leads to Che-1/AATF degradation following apoptotic DNA damage has been recently elucidated in two papers from De Nicola et al. (2007). They showed that upon apoptotic DNA damage the kinase HIPK2 directly interacts with Che-1/AATF and phoshorylates it at residue T144. This phosphorylation allows a conformational change mediated by the prolyl isomerase Pin1, which in turn promotes the interaction with ubiquitin ligase HDM2, thereby promoting Che-1/AATF ubiquitylation and proteasomal degradation. In agreement with these results, Che-1/AATF overexpression interferes with HIPK2 induced apoptosis, while failure in Che-1/AATF degradation upon apoptotic stimuli is associated with reduced cell death (De Nicola et al., 2007, 2014).

As described above, Che-1/AATF strongly affects p53 functions upon DNA damage by activating its transcription, promoting p53 dependent growth arrest and inhibiting p53 dependent apoptosis. Notably, this protein has also a strong impact on the activity of the mutant forms of p53 (mtp53), which are associated with almost 50% of cancer cases (Freed-Pastor and Prives, 2012). Indeed, Che-1/AATF is required for mtp53 transcription and its depletion induces apoptosis, without involving any other stimuli, in several cancer cell lines carrying mtp53. This event is the result of a simultaneous reduction of mtp53 level and activation of pro-apoptotic genes, such as *Puma* and *Noxa*, by tumor suppressor protein p73. In more detail, in the presence of mtp53, Che-1/AATF depletion induces endogenous checkpoint activation that leads to stabilization of the transcription factor E2F1, which in turn, activates p73. Inline with Che-1/AATF’s ability to modulate checkpoint activation, Affymetrix microarray experiments have revealed that this protein regulates the expression of genes involved in DNA repair (Bruno et al., 2010).

### Cellular Stress

**FIGURE 1:**
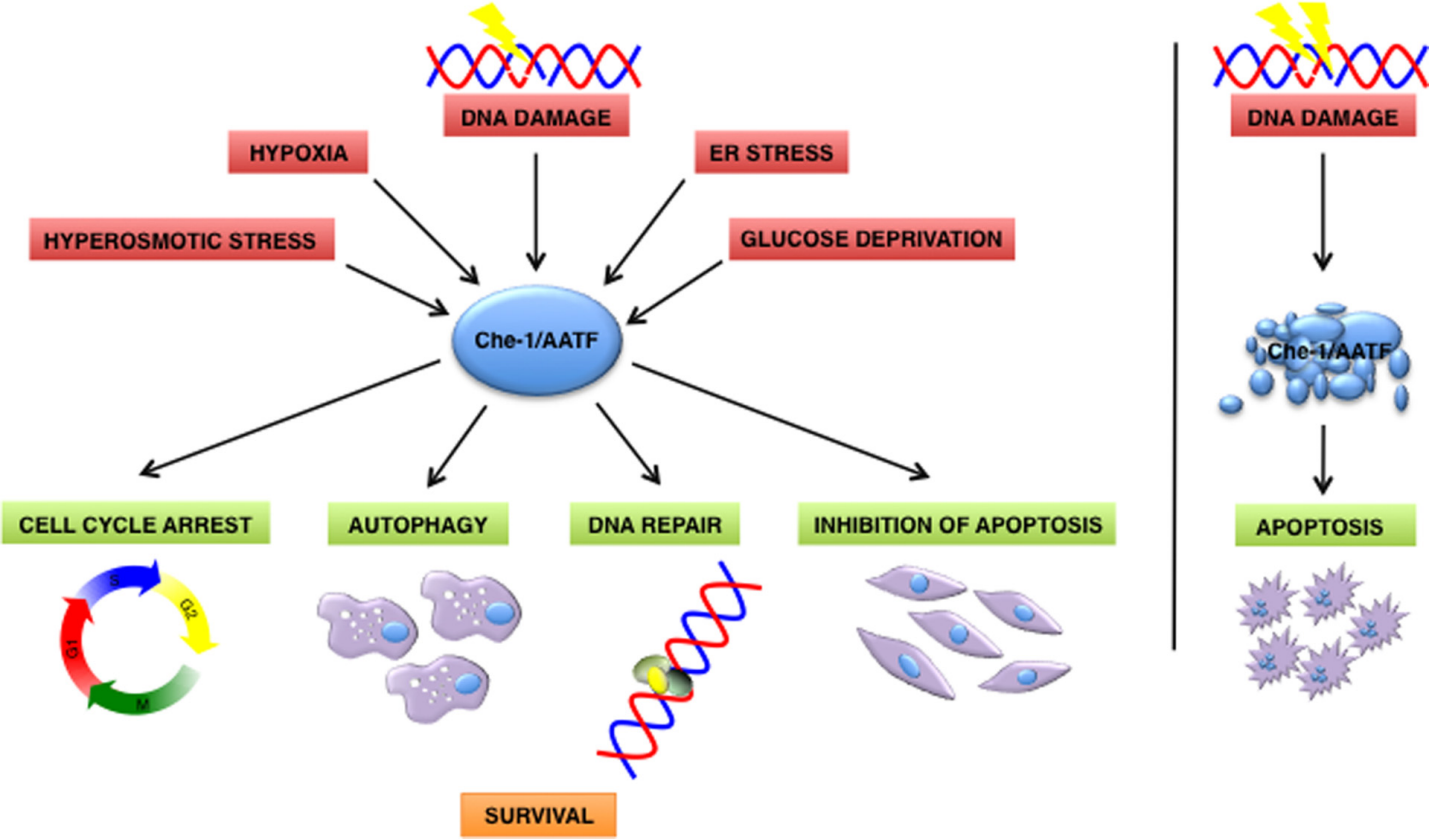
**Che-1/AATF is a central mediator of the cellular response to different types of stress**. In response to DNA damage and cellular stress Che-1/AATF promotes cell survival by inducing cell cycle arrest, autophagy, DNA repair and inhibition of apoptosis. Otherwise if DNA damage is too severe Che-1/AATF is degradated and cells undergo apoptosis. ER, endoplasmatic reticulum.

Over the last few years, several pieces of evidence indicate that Che-1/AATF participates in the cellular response to different types of stress, other than DNA damage (**Figure 1**). For example, hyperosmotic stress can activate Che-1/AATF by inducing MK2-mediated phosphorylation (Höpker et al., 2012).

Ishigaki et al. (2010) indicated Che-1/AATF as a component of the unfolded protein response (UPR), an adaptative mechanism activated by endoplasmatic reticulum (ER) stress whose function is to restore ER homeostasis or induce apoptosis if stress cannot be resolved. Che-1/AATF is induced upon ER stress and promotes cell survival by activating transcription on the serine/threonine kinase AKT1, through directly interacting with transcription factor STAT3. Indeed, ectopic expression of Che-1/AATF protects cells from ER stress mediated apoptosis whereas its depletion increases the percentage of apoptotic cells after induction of ER stress (Ishigaki et al., 2010).

More recently, it has been demonstrated that Che-1/AATF protects cells from apoptosis induced by ionizing radiations (IR), hypoxia, or glucose deprivation by inducing autophagy, a self degradative process essential for maintaining cellular homeostasis that allows cells to survive under metabolic stress. In particular, Che-1/AATF inhibits the activity of the kinase mTOR, a central regulator of autophagy, by activating the transcription of its inhibitors Redd1 and Deptor. In agreement with these results, Che-1/AATF depletion decreases autophagy induction after stress, thus leading to apoptosis (Desantis et al., 2015b). Interestingly, it has been recently reported that inhibition of serum deprivation induced autophagy by resveratrol reduces phosphorylation of Che-1/AATF at residues S316, S320, and S321; however, the kinases responsible of these modifications are still unknown (Alayev et al., 2014).

## Che-1/AATF as a Putative Therapeutic Target in Cancer

Overall, the observations described above strongly indicate that Che-1/AATF plays an important role in many aspects of cancer biology. Indeed, this protein is not only involved in cell cycle progression and in protecting cancer cells from apoptosis induction, but also plays a role in controlling autophagic response and ER stress, appearing to be able to sustain the survival of tumor cells (Desantis et al., 2015b). Moreover, Che-1/AATF deeply affects the activity of p53, by both modulating wild type p53 target specificity and supporting the “gain of function” of the mutated forms of this oncosuppressor (Bruno et al., 2010; Desantis et al., 2015a). However, a screening for Che-1/AATF mutations in 121 breast cancer families has highlighted that no mutations in Che-1/AATF coding sequence can be associated with cancer predisposition (Haanpää et al., 2009). On the other hand, several studies suggest that dysregulation in Che-1/AATF level inside cells could be relevant for the transformation process. In fact, this protein has been found upregulated in several leukemia cell lines and in patients with chronic lymphocytic leukemia (Kaul and Mehrotra, 2007; Bacalini et al., 2012). In addition, *Che-1/AATF* gene was amplified in neuroblastoma patients and increased Che-1/AATF expression levels were associated with poor prognosis and reduced survival (Höpker et al., 2012). Consistent with these observations, Che-1/AATF depletion was shown to enhance the cytotoxic effect of DNA-damaging chemotherapy both *in vitro* and *in vivo* and to induce apoptosis of cancer cells carrying mtp53 (Bruno et al., 2006, 2008, 2010; Höpker et al., 2012). All these findings strengthen the notion that Che-1/AATF could be considered a valid target for novel anticancer therapeutic approaches. Unfortunately, so far no compounds able to inhibit Che-1/AATF activity have been identified. However, future efforts focused on understanding the mechanism of action of Che-1/AATF and the characterization of the pathways implicated in its regulation will provide useful indications towards developing specific inhibitors for this protein.

## Concluding Remarks and Open Questions

Much has been learned about Che-1/AATF functions in the years following its identification but a great deal remains to be unveiled.

One question that needs to be addressed is its role in DNA repair. Bruno et al. (2010) have shown that Che-1/AATF expression is necessary for proper repair of damaged DNA, but how this action is exerted is still not entirely understood. The ability of Che-1/AATF to regulate the expression of genes involved in DNA repair is definitely part of this process but other mechanisms may participate. At the structural level, one of the main features of Che-1/AATF is the presence of an extremely acidic domain at its N-terminal region, which in other transcription factors has been associated with chromatin remodeling properties (Hu et al., 1999; Tumbar et al., 1999). Moreover, Che-1/AATF has been found in histone acetyltransferase complexes through its interaction with the transcriptional co-activator ADA3 (Zencir et al., 2013) and it has the ability to induce local histone hyperacetylation by displacing HDAC1 from transcription factors pRb and Sp1 (Bruno et al., 2002; Di Padova et al., 2003). Based on these observations, one could speculate that Che-1/AATF participates in the DNA repair process by regulating the chromatin state and increasing its accessibility. If so, a new scenario for Che-1/AATF functions will be open since chromatin remodeling plays a fundamental role in replicative and transcriptional controls. Indeed, Che-1/AATF could participate in the regulation of gene expression by regulating chromatin structure at specific gene loci where it is recruited by its interaction with both transcription factors and transcriptional machinery.

Furthermore, since Che-1/AATF has already been appointed as a nucleolar stress sensor (Ferraris et al., 2012), it will be interesting to further investigate its involvement in the nucleolar stress response that monitors and maintains ribosome biogenesis and nucleolar integrity. This pathway has a crucial role in maintaining cellular homeostasis and it has been demonstrated that nucleoli disruption leads to activation of p53 in absence of DNA damage (Rubbi and Milner, 2003). Moreover, transformed cells undergo p53 mediated senescence, autophagy, and apoptosis in response to nucleolar perturbation by inhibition of ribosomal RNA synthesis (Woods et al., 2014). Indeed, in the last few years nucleolus and ribosomal gene expression are emerging as new exciting targets for cancer therapy and RNA polymerase I inhibitors are currently entering phase I clinical trials (Quin et al., 2014). In this context, it will be fundamental to explore the possibility that Che-1/AATF plays a role in ribosome biogenesis itself. This idea is supported by the observations that a mouse embryo mutant for *Traube* shows a decrease in the number of ribosomes and *Drosophila* Che-1/AATF mutants arrest the development of the egg chamber at the same stage as the mutants affecting the synthesis of ribosomes, namely when the massive growth starts and cells need to synthesize ribosomes to trigger this growth (Jagut et al., 2013).

Answering these questions will shed further light on additional aspects of Che-1/AATF functions and likely contribute to identifying possible therapeutic approaches involving this protein.

## Conflict of Interest Statement

The authors declare that the research was conducted in the absence of any commercial or financial relationships that could be construed as a potential conflict of interest.
